# Inorganic process for wet silica-doping of calcium phosphate†

**DOI:** 10.1039/d1ra00288k

**Published:** 2021-03-29

**Authors:** Yuki Sugiura, Kodai Niitsu, Yasuko Saito, Takashi Endo, Masanori Horie

**Affiliations:** Health and Medical Research Institute, National Institute of Advanced Industrial Science and Technology (AIST) Kagawa 761-0395 Japan yuki-sugiura@aist.go.jp; Department of Material Science and Engineering, Kyoto University Kyoto 606-8501 Japan; Research Institute for Sustainable Chemistry, National Institute of Advanced Industrial Science and Technology (AIST) Hiroshima 739-0046 Japan

## Abstract

Silica is not only a biocompatible trace element but also an essential element for bone formation and metabolism. Therefore, it is often doped into bioceramics such as calcium phosphate and calcium carbonate for enhancing biomaterial ability. Heretofore, organic silica materials are employed as silica sources, but the residual organic matter is a significant drawback in biomaterial applications. Therefore, in this study, we introduce a one-pot inorganic synthesis method for the formation of silica-doped octacalcium phosphate (OCP) using Na_2_SiO_3_ as the silica source. Silica was intercalated into the OCP unit lattice, replacing its hydrous layer structure, and then a layer-by-layer structure of apatite and silica was formed. Furthermore, by immersing the fabricated silica-doped OCP into suitable solutions, both silica-doped hydroxyapatite and carbonate apatite were fabricated through a one-step inorganic processes.

## Abstract 2

We introduced a one-pot synthesis method for silica doping of calcium phosphate. Silica easily incorporated into OCP interlayer optimizing Na_2_SiO_3_ concentrations.

## Graphical abstract

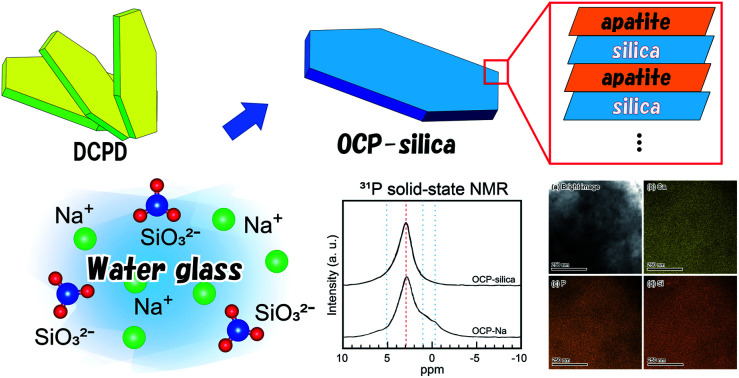

## Introduction

Since the 1960s, silica (SiO_4_^4−^) has been known to stimulate and enhance bone cell activities and upregulate the bone remodelling process.^1–3^ Especially, silica enhances the activity and purification of osteoblasts as reinforcing alkali phosphatase and type-I collagen generation and inhibits the activity of osteoclasts.^4–6^ Moreover, silica-doped biomaterials, due to their excellent biocompatibility, are considered attractive for bone substitutes.

Various works have investigated silica-doped biocompatible ceramics, such as calcium carbonate and calcium phosphate.^7–10^ For example, silica-doped vaterite, a metastable phase of calcium carbonate, has been found to significantly upregulate osteoblast activities such as alkali phosphatase generation and viability.^7,11^ However, silica-doping processes of calcium carbonate and calcium phosphates based on wet-chemical systems have significant drawbacks. Although organic silica materials such as tetraethyl orthosilicate (TEOS) and hexamethyldisilane have been employed as silica sources for doping *via in situ* hydrolysis, the residual organic matter from silica sources might exhibit toxicity.^7,8,11,12^

Octacalcium phosphate [OCP: Ca_8_H_2_(PO_4_)_6_·5H_2_O] exhibits excellent biocompatibility and can function as a bone replacement material through bone remodeling.^13–16^ In addition, OCP functions as a precursor material for various calcium phosphates, such as apatite, through solid–solid phase conversion processes.^17–19^ Therefore, silica-doped OCP can lead to the introduction of various silica-doped calcium phosphates.

A technique for fabricating silica-doped OCP in sodium silicate (Na_2_SiO_3_) solutions would be valuable for the silica-doping of calcium phosphate; therefore, in this study, we investigated silica-doped OCP formed through hydrolysis process in inorganic Na_2_SiO_3_ solutions.

## Material and methods

### Materials

All reagents were purchased from FUJI Film Wako Pure Chem Inc., Japan. The compounds Na_2_SiO_3_ and (NH_4_)_2_CO_3_ were dissolved in distilled water to form solutions. The mother experimental solutions were prepared as 5.0 mol L^−1^ Na_2_SiO_3_ and 2.0 mol L^−1^ (NH_4_)_2_CO_3_; then, they were diluted with distilled water for solution preparation.

A 2.39 g sample of dicalcium hydrogen phosphate dihydrate (DCPD: CaHPO_4_·2H_2_O, 14 mmol) was immersed in 20 mL of the abovementioned solutions at 60 °C for 1 day. The initial and final pH values of the solutions were recorded at room temperature using a pH electrode (LAQUA ToupH 9615S-10D with pH meter D-72, Horiba Co., Kyoto, Japan). The treated samples were washed with distilled water several times to remove the residual solutions and then placed in a drying oven at 60 °C for several hours.

### Characterization

The crystallographic information about the samples was obtained *via* X-ray diffraction (XRD: MiniFlex600, Rigaku Co., Japan) at an acceleration voltage and amplitude of 40 kV and 15 mA, respectively. The diffraction angle was continuously scanned over 2*θ* values ranging from 3° to 70° at a scanning rate of 5°/min for characterization and from 2° to 12° at a scanning rate of 1° min^−1^ for crystallographic parameter analysis.

The chemical vibration scheme of the samples was characterized *via* Fourier-transform infrared (FTIR) spectroscopy (VERTEX, Bruker Optics Co., USA) using a triglycine sulfate detector (128 scans, resolution of 4 cm^−1^) with an attenuated total reflection prism made of diamond. The atmosphere was the background for conducting the measurements.

The ^31^P chemical shifts of the samples were determined *via* solid-state nuclear magnetic resonance (NMR) spectroscopy (Varian FT-NMR, 400 MHz, Agilent Technologies Co., USA), with a resonance frequency of 161.8 MHz for ^31^P. For the measurements, cross-polarization magic-angle-spinning (CP-MAS) ^31^P NMR spectroscopy was performed under a CP-MAS rate of 10 kHz. An Agilent 4 mm T3 CP-MAS HXY solid probe and zirconia rotors were used. The sample weight was ∼0.02 g, and the contact time for the ^31^P CP-MAS measurements was 5 ms, with an acquisition time of 10 ms and relaxation delays of 40 s for each measurement interval. The number of repetitions per measurement was 400. The ^31^P chemical shift of (NH_4_)H_2_PO_4_ was used as an external reference (*δ* = 1.0 ppm).

The fine structure of the samples was assessed *via* field-emission scanning electron microscopy (FE-SEM: JSM-6700F, JEOL Co., Japan) at an acceleration voltage of 5 kV; the samples were sputter-coated using Os to prevent surface-charge accumulation. The fine atomic distribution of the samples was observed *via* scanning transmission electron microscopy-energy-dispersive X-ray spectroscopy (STEM-EDX, JEM-ARM200F, JEOL Co., Japan) at an acceleration voltage of 200 kV.

The Ca and P(PO_4_) (and Na) contents of the samples were measured *via* inductively coupled plasma-atomic emission spectroscopy (ICP-AES: 5110VDV, Agilent Technology Co., Japan) after the samples were dissolved in 2% HNO_3_ solution.

The thermal behaviour of the samples was determined *via* thermogravimetry-differential thermal analysis (TG-DTA; Thermo-Plus, TG8110, Rigaku Co., Japan). The weight loss ratio around 150 °C was defined as the hydrous layer consisting of HPO_4_ and OH decomposition.

The Si content of the samples was measured *via* X-ray fluorescence spectroscopy (XRF: SEA2210, SII Nano Technology Co., Japan) at an acceleration voltage of 15 kV under vacuum conditions. The Si/Ca ratio was calibrated using a disk-shaped mixture of hydroxyapatite-SiO_2_ sintered blocks.

## Results and discussions

**Fig. 1:**
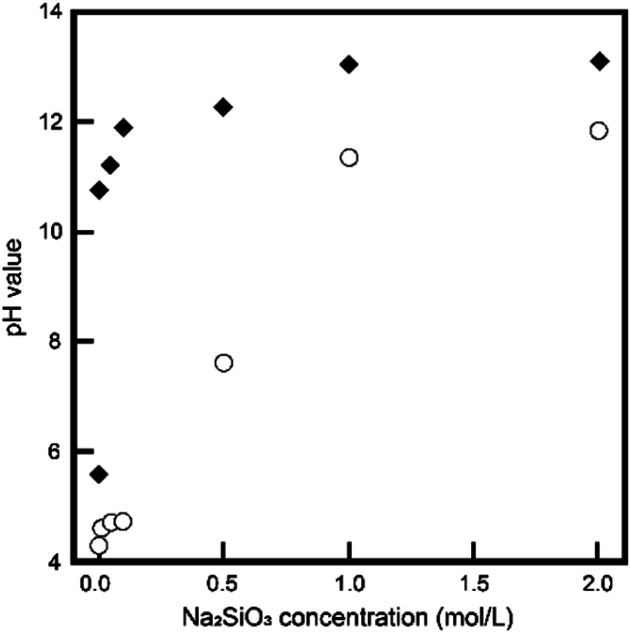
The initial (◆) and final (〇) pH values, respectively, of the reaction solutions with different Na_2_SiO_3_ concentrations.

In this study, we employed the hydrolysis of DCPD, an acidic calcium phosphate, in Na_2_SiO_3_ solutions, a strongly basic solution. The pH of the reacting solutions is an important factor for the phase conversion process, because each phase of the calcium phosphate formation was greatly dependent on the pH. Fig. 1 shows the initial and final pH values of reacting solutions. With increasing Na_2_SiO_3_ concentration, the initial pH values of reacting solutions increased to reach about 13.5; likewise, the final pH values increased to reach about 12.0. When the Na_2_SiO_3_ concentration was above 1 mol L^−1^, the pH values of the reacting solutions were between 12.0 to 13.5, as apatite preferentially formed under the solution conditions.

**Fig. 2:**
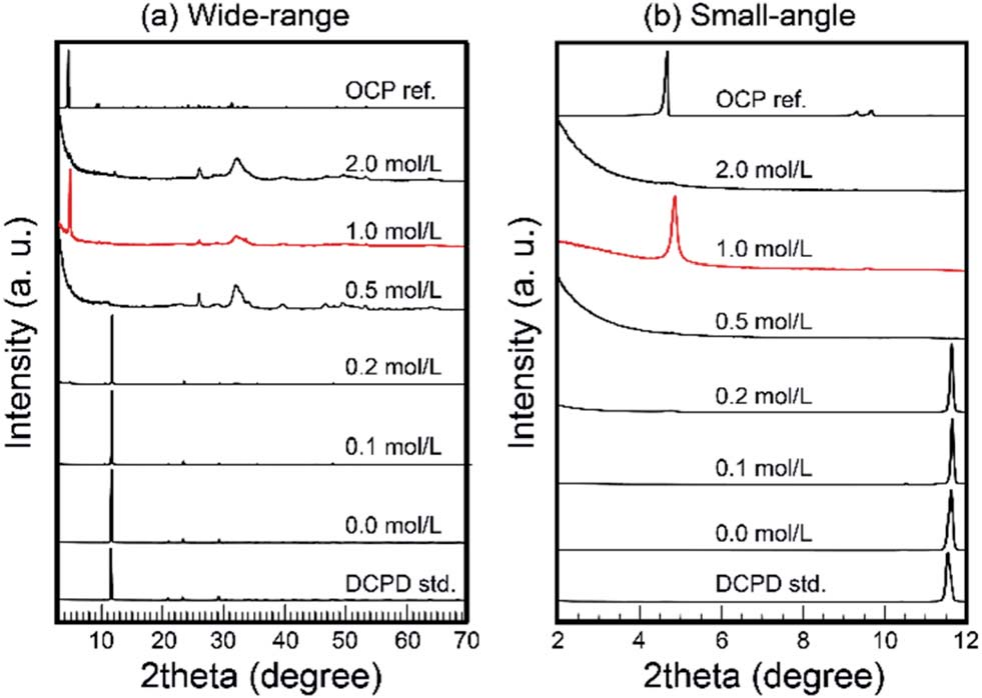
Wide-range (a) and small-angle (b) XRD patterns of samples treated under various Na_2_SiO_3_ concentrations. Note: in the case of 1 mol L^−1^ Na_2_SiO_3_, the XRD pattern of sample exhibited OCP pattern.

Then, the treated samples were characterized *via* XRD. Fig. 2 shows the XRD patterns of treated samples. In the cases of low Na_2_SiO_3_ concentration (<0.2 mol L^−1^), residual DCPD was the main phase. In the case of 0.5 mol L^−1^ Na_2_SiO_3_, the treated samples transformed into monophasic apatite. Then, in the case of 1.0 mol L^−1^ Na_2_SiO_3_, a strong peak at ∼4.7°, likely OCP d(100) peak, occurred with large parts of conventional OCP peaks. Interestingly, in the cases of 2.0 mol L^−1^ Na_2_SiO_3_, the strong peak at ∼4.7° was absent, and the samples appeared to transform into monophasic apatite.

In the case of 1 mol L^−1^ Na_2_SiO_3_, the XRD pattern of the samples was similar to that of OCP. However, several ordinal peaks such as d(200) and d(110) at ∼9.2° and ∼9.7°, respectively, were absent. In addition, the crystallinity of the obtained samples was too low to enable the effective characterization of their crystal structure. Therefore, it was considered that layered composites consisted of silica-based clay minerals.

**Fig. 3:**
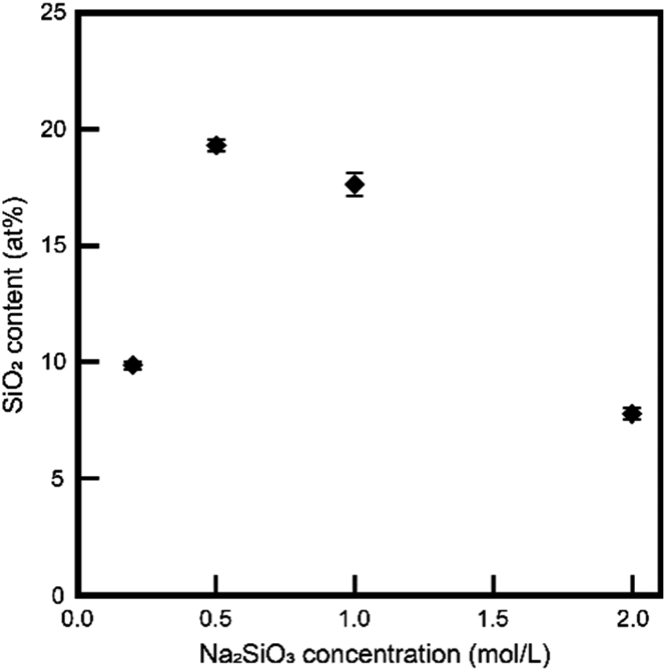
Si contents of samples.

We focused on the chemical compositions of samples. As the first step, we evaluated the silica contents of samples *via* XRF measurements. Fig. 3 shows the silica contents of the samples. We did not measure samples below 0.2 mol L^−1^ Na_2_SiO_3_ because of the large residual initial DCPD. Interestingly, the silica contents of the samples and the Na_2_SiO_3_ concentrations of treated solutions were inversely proportional. Through ICP-AES measurements, the chemical composition of treated samples in 1 mol L^−1^ Na_2_SiO_3_ was determined as Ca_8_Na_1.12_H_6.4_(PO_4_)_4_(SiO_4_)_2.88_·*n*H_2_O for a pure substance.

**Fig. 4:**
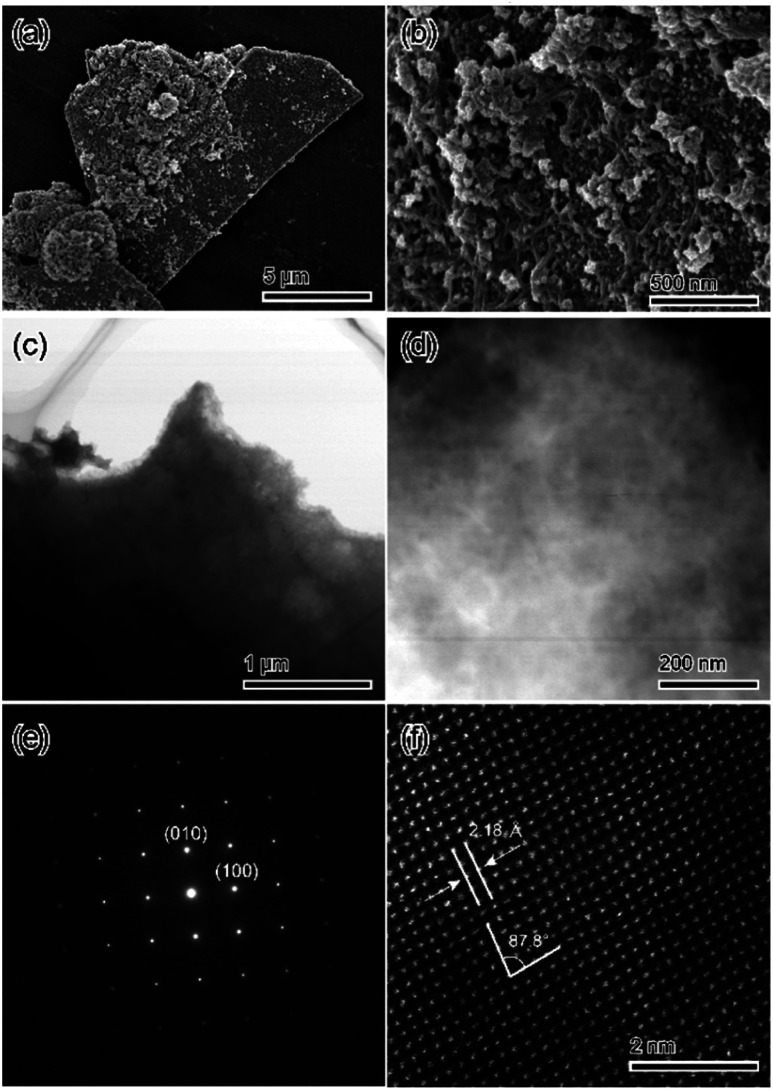
Fine structure observation results of the sample treated in 1 mol L^−1^ Na_2_SiO_3_ solutions: (a and b) whole and surface structures; (c and d) inner structure and (e and f) its crystallographic analysis.

The morphologies of samples were identified *via* scanning electron microscopy (SEM) to confirm if the obtained materials were a pure substance or a mixture. Fig. 4 shows the SEM micrographs of treated samples. The microparticles of the treated samples exhibited plate-like crystal morphology, which is representative of DCPD crystals. Therefore, we consider that the treated samples have a pseudomorphic relationship with DCPD (Fig. 4a). In the magnified image, the surface of the treated samples consisted of closely packed ∼10 nm particles (Fig. 4b). The fine inner structure including the crystal structure of samples was determined *via* transmission electron microscopy (TEM). The inner structure of the samples was essentially uniform (Fig. 4c and d). Furthermore, the selected-area diffraction pattern and the STEM-high-angle annular dark-field imaging results of the treated samples indicated that the samples had enough sizes of single-crystal domains (Fig. 4e and f).

**Fig. 5:**
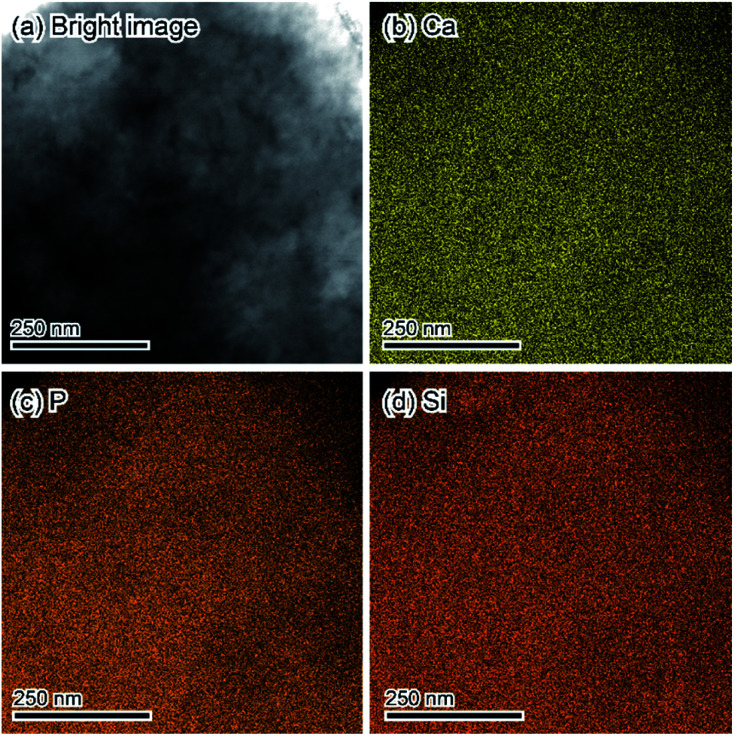
STEM-EDX mapping results of the sample treated in 1 mol L^−1^ Na_2_SiO_3_ solutions corresponding to Fig. 4d. (a) Bright image. (b) Ca. (c) P. (d) Si.

The fine chemical composition distributions of treated samples were evaluated *via* STEM-EDX. Fig. 5 displays the STEM-EDX mapping images of treated samples. All observed elements; Ca, P, and Si, were uniformly distributed.

Based on the bulk and microscopic evaluation, we conclude that the pure substance consisted of Ca, PO_4_, and silica with an OCP-like crystal structure was formed when DCPD was treated in 1 mol L^−1^ Na_2_SiO_3_ solution. Thus, we designate the treated samples as OCP-silica.

It seems that the results of XRD patterns and TEM micrographs of OCP-silica did not coincide. This phenomenon could be described the unique crystal structure of OCP composite as the laminated structure of well-arranged 2D crystal structure. When observed along the vertical direction of lamination, well-arranged fine structure could be seen in spite of the bulk layer structure. Furthermore, the results of spectroscopic and STEM observation also supported this consideration.

**Fig. 6:**
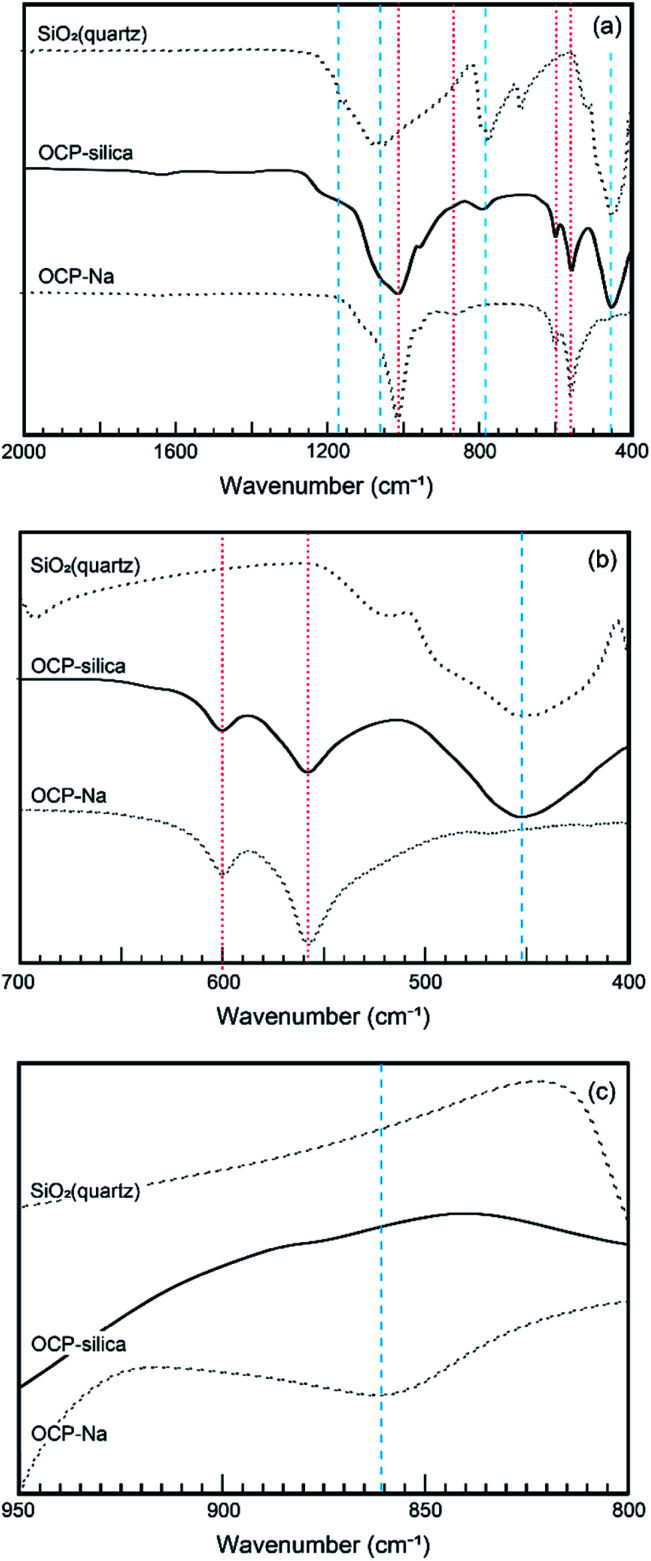
FT-IR spectra of OCP-silica and reference materials (OCP-Na and SiO_2_). (a) Wide-range. (b) *P*6 PO_4_ region. (c) *P*5 PO_4_. Red dotted lines and blue broken lines corresponded to silica and calcium OCP, respectively.

Furthermore, we evaluated the physicochemical properties of OCP-silica. First, we focused on which sites were substituted by silica in the OCP crystal structure. Normally, six different states of PO_4_ (*P*1 to *P*6) occurred in the OCP unit lattice. Each PO_4_ state (*P*1 to *P*6 PO_4_) of the OCP unit lattice could be determined through spectroscopic analysis based on the OCP low symmetry crystal structure (*P*1̄). Fig. 6 shows the FTIR spectra of OCP-silica with SiO_2_ and OCP-Na to facilitate comparison. In the case of OCP-silica, PO_4_ and HPO_4_ bands were observed, which are normally observed in OCP, with silanol bands 455 cm^−1^ and, around 1100 cm^−1^ to 1300 cm^−1^. Considering to characteristic bands of quarts (455 cm^−1^), the crystallite size of silica was smaller than 3 μm.^20^ Moreover, we observed little *P*5 PO_4_ band in OCP-silica, which is the root of the hydrous layer.

**Fig. 7:**
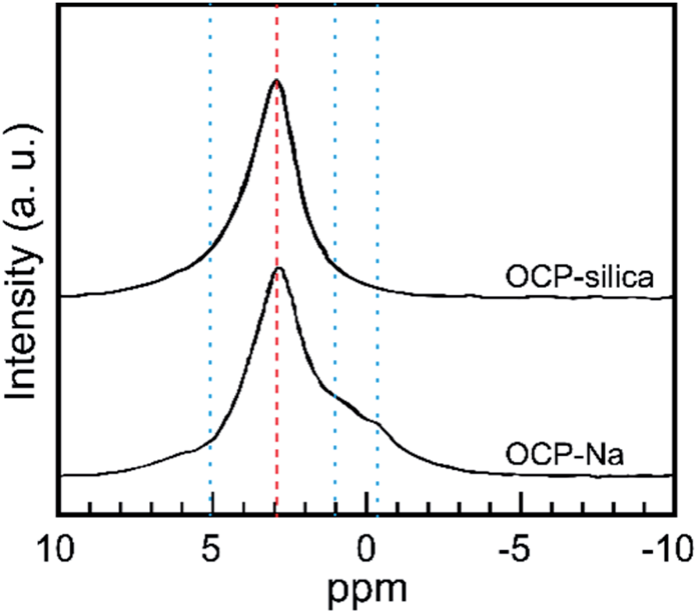
^31^P solid-state NMR spectra of OCP-silica and OCP-Na. Red broken line: state of apatite layer, blue dotted line: state of hydrous layer.

Furthermore, we evaluated the state of *P*6 PO_4_, the centerpiece of the hydrous layer, *via* solid-state NMR, as it could not be well evaluated through FTIR analysis. Fig. 7 shows the ^31^P solid-state NMR spectra of OCP-silica and OCP-Na to facilitate comparison. Based on the FTIR and solid-state NMR results, little PO_4_ bands corresponding to hydrous layers (*P*5 and *P*6 PO_4_) were observed.

**Fig. 8:**
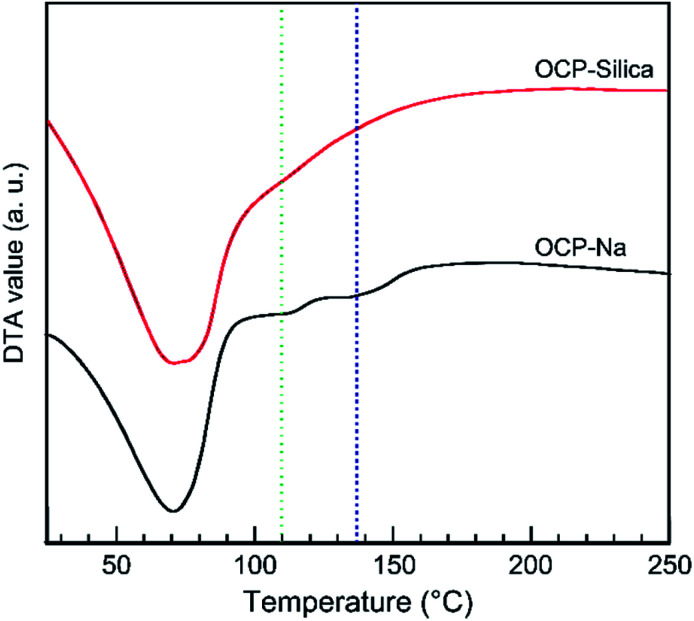
DTA curve of OCP-silica and OCP-Na. Green and blue broken lines were indicated the collapse of the OCP hydrous layer of OCP.

Through thermal analysis, we investigated the thermal stability of samples. Especially, for OCP, we determined the decomposition temperature and behaviour of the hydrous layer. Fig. 8 shows the DTA curves of OCP-silica and OCP-Na to facilitate comparison. In the case of OCP-silica, the decomposition of the OCP hydrous layer was hardly observed. Thus, we conclude that silica could be substituted in the OCP hydrous layer.

**Fig. 9:**
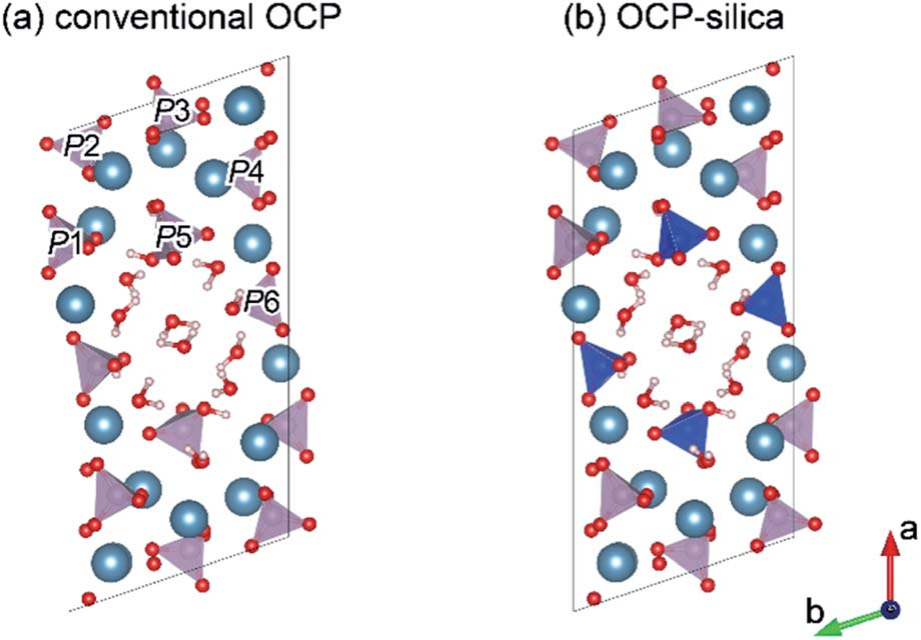
Schematic illustrations of conventional OCP (a) and OCP-silica (b) crystal structure drawn by VESTA3 programme based on ref. 30 and ^31^ Each phosphates were labeled as *P*1 to *P*6. Blue tetrahedron indicated SiO_4_^4−^.

By tailoring Ca, PO_4_, and silica, we could synthesize OCP-silica through a one-step process using only inorganic composites. We concluded that the silica contents of the samples did not coincide with the Na_2_SiO_3_ concentrations due to the two factors of solution pH and molar ratio of Ca and PO_4_ + silica. Several previous studies indicate that OCP was likely formed in PO_4_ rich solutions.^21–23^ This study suggests that silica also played a similar role of PO_4_ for OCP formation. At further concentrated conditions, high alkalinity induced apatite formation. Based on the results of XRD, FTIR, and solid-state NMR, we roughly drew a schematic illustration of OCP-silica crystal structure in Fig. 9. Briefly, silica molecules replaced the HPO_4_ ions of hydrous layer of OCP unit lattice.

Then, we evaluated the properties of the fabricated OCP-silica, focusing more on the phase-conversion property. When the OCP was immersed in hot water or placed under hydrothermal conditions, it converted to apatite *via* direct solid–solid phase transformation.^18,19,24^ In addition, when the OCP was heated under dry conditions, it converted to tricalcium phosphate (TCP) through the collapse of the OCP structures. Fig. S1† shows the XRD patterns of OCP-silica samples treated in water and in (NH_4_)_2_CO_3_ containing solutions. The treated OCP-silica samples transformed into monophasic apatite regardless of the (NH_4_)_2_CO_3_ concentration. Moreover, FTIR measurements indicate that the obtained apatite samples contained CO_3_ as carbonate apatite [CO_3_Ap: Ca_10−*a*_(PO_4_)_6−*b*_(CO_3_)_c_(OH)_2−*d*_] (Fig. S2†). With increasing (NH_4_)_2_CO_3_ concentration in the treated solutions, the CO_3_ content of samples increased, while the silica content slightly decreased. Then, silica-substituted apatite with/without CO_3_ could be fabricated from OCP-silica *via* a simple solution-immersion process (Fig. S3†).

We also evaluated the phase evolution of OCP-silica during dry heat processes. Fig. S4† shows the XRD pattern of samples subjected to dry heat processes. With increasing temperature, OCP-silica converted to monophasic hydroxyapatite (HAp) at 200 °C. Then, with further temperature increase to 600 °C, cristobalite (SiO_2_) peaks were observed with HAp peaks. Above 1000 °C, samples were converted to TCP and cristobalite. Moreover, during heat treatment at above 600 °C, granular particles of 10–30 nm formed on the OCP-silica surface, which coincides with the XRD results (Fig. S5†).

The results indicate that during the DCPD hydrolysis process in a solution of suitable Na_2_SiO_3_ concentration, Ca, PO_4_, and silica assembled as the OCP structure. The results of STEM-EDX mapping and ^31^P solid-state NMR showed that silica was intercalated into the OCP hydrous layer, and then a layer-by-layer structure of apatite-silica was formed. In addition, similar to the conventional OCP, OCP-silica could be converted to apatite-containing silica through a simple solution-immersion process.

Much attention and cost have been channelled toward safely substituting and doping silica to biomaterials. The proposed method is simple and safe, as no organic materials are needed for silica substitution; thus, harmful residual organic matter is avoided.

**Fig. 10:**
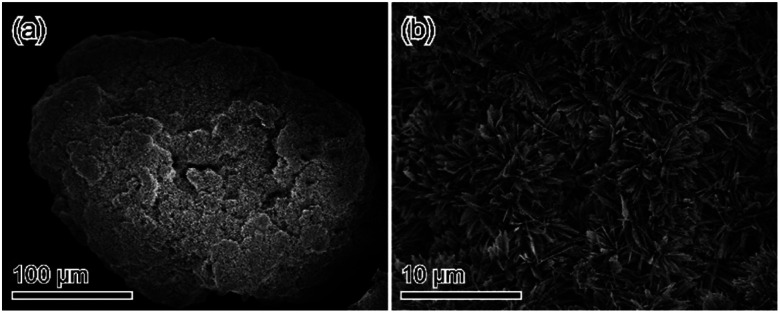
SEM micrograph of DCPA granules treated in 1 mol L^−1^ Na_2_SiO_3_: (a) low-magnification image; (b) magnified image.

For biomaterial applications, especially as bone substitutes, at least granule size (>100 μm) is needed.^25–27^ The proposed process enables the application of such granules, as calcium hydrogen phosphate anhydrate [DCPA: CaHPO_4_], the anhydrate phase of DCPD, has excellent formability that allows the formation of granule-shaped materials.^16,28,29^ When DCPA granules (100–250 μm) were immersed into Na_2_SiO_3_ solution, OCP-silica granules preserved precursor whole shapes could be obtained (Fig. 10 and S6†). Furthermore, when the fabricated OCP-silica granules were immersed into (NH_4_)_2_CO_3_ solutions, they converted to HAp, or CO_3_Ap maintained their whole shapes (Fig. S7–S9†). Thus, this method can overcome the bottleneck of safe silica-doping using silica-based materials.

## Conclusion

In conclusion, OCP-silica was fabricated from soluble calcium phosphate through the immersion of DCPD into Na_2_SiO_3_ solution. Silica was intercalated into the OCP hydrous layer, replacing the hydrous structure. When OCP-silica was immersed in aqueous solution, silica-substituted apatite (both HAp and CO_3_Ap) could be fabricated *via* one step.

## Author contributions

Y. Sugiura and M. H. did setup of experimental design and performed experiments. K. N. performed TEM and STEM observations. Y. Saito and T. E. measured solid-state NMR of samples. Y. Sugiura wrote a manuscript.

## Conflicts of interest

There are no conflicts to declare.

## Supplementary Material

RA-011-D1RA00288K-s001
