# Supplementary material for: Inorganic process for wet silica-doping of calcium phosphate
Source: RSC Adv. 2021 Mar 29;11(20):12330–5. doi: 10.1039/d1ra00288k (PMC8696845; doi:10.1039/d1ra00288k)
Supplement: RA-011-D1RA00288K-s001 [file RA-011-D1RA00288K-s001.pdf]

Electronic Supplementary Material (ESI) for RSC Advances.
This journal is © The Royal Society of Chemistry 2021

Supporting Information: Inorganic process for wet-silica doping of calcium phosphate

Yuki Sugiura, Kodai Niitsu, Yasuko Saito, Takashi Endo, Masanori Horie

Fabrication of silica substituted apatite from OCP-silica

First, 0.4 of OCP-silica powder was immersed in 20 mL of 0.0–2.0 mol/L $(NH_4)_2CO_3$ solution at 80 °C for three days. The treated OCP-silica powders were washed with distilled water several times and then oven-dried at 80 °C overnight.

Fabrication of OCP-silica granules.

Precursor DCPA granules were fabricated as analogs of the dental brushite cement setting reaction. Calcium dihydrogen phosphate hydrate and β-TCP were mixed at a 1:1 molar ratio using an agate mortar and pestle. Then, ~1g of the mixture was placed into a rotary pan-type granulator (PZ-01R, As One Co., Japan) with stirring at 40 rpm. Thereafter, approximately 1.0 mL of distilled water was added as a spray to obtain granules of acidic calcium phosphate. The set mixture was continuously stirred for 10 min and then placed in a dry oven at 40 °C overnight. The dried set mixture was separated using an automatic sieve of 250–500 μm.

Then, ~1.0 g of DCPA granules was immersed in 20 mL of 1 mol/L Na_2SiO_3 at 60 °C for two days. The treated granules were then washed with distilled water several times and placed in a dry oven at 40 °C to remove residual moisture.

Fabrication of apatite–silica granules from OCP-silica granules.

First, ~0.4 g of fabricated OCP-silica granules was immersed in 20 mL of 0.0–2.0 $(NH_4)_2CO_3$ solutions at 80 °C for three days. The treated granules were then washed with distilled water several times and placed in a dry oven at 40 °C to remove residual moisture.

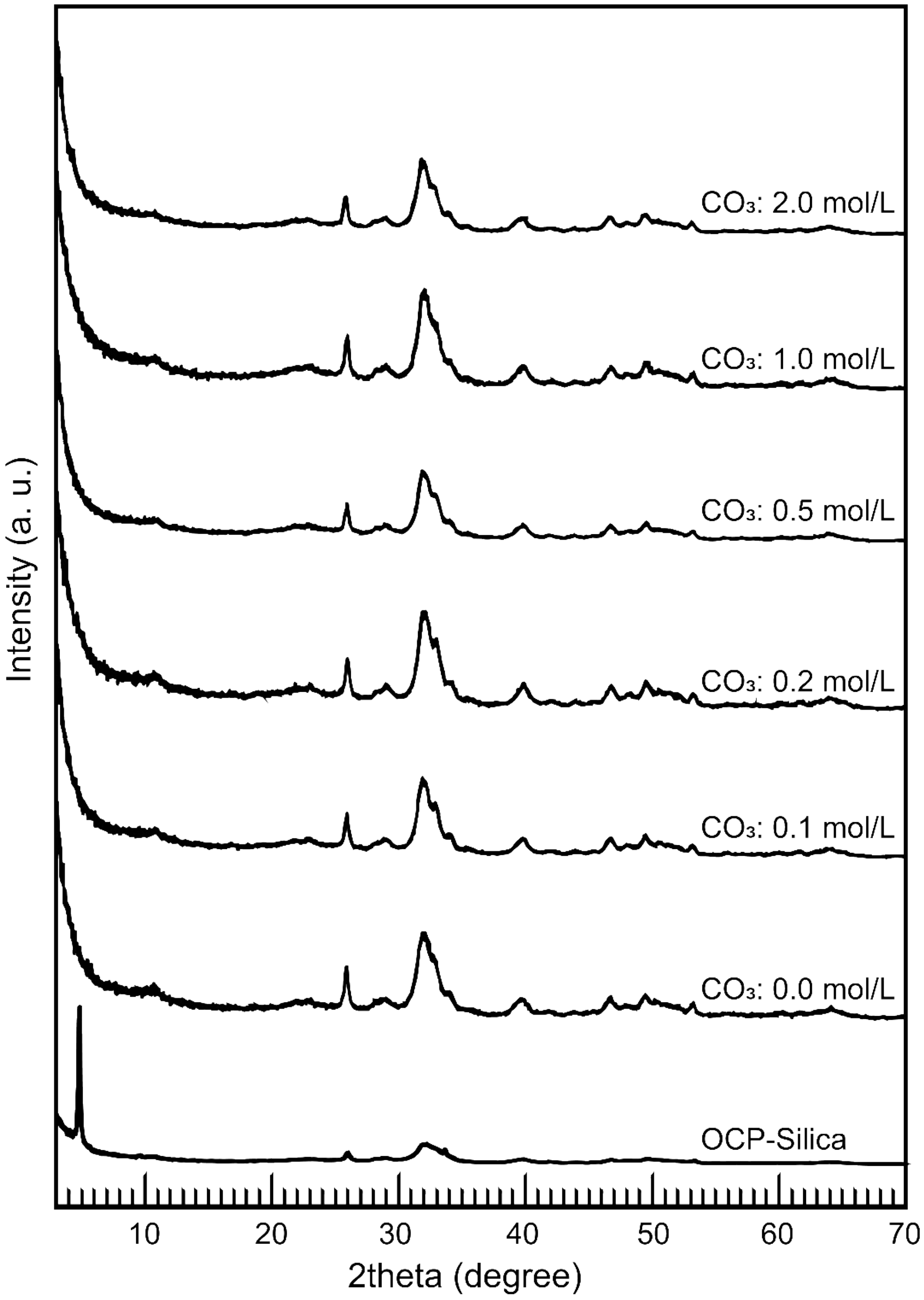

Figure S1. XRD patterns of OCP-silica treated in various $(NH_4)_2CO_3$ solutions.

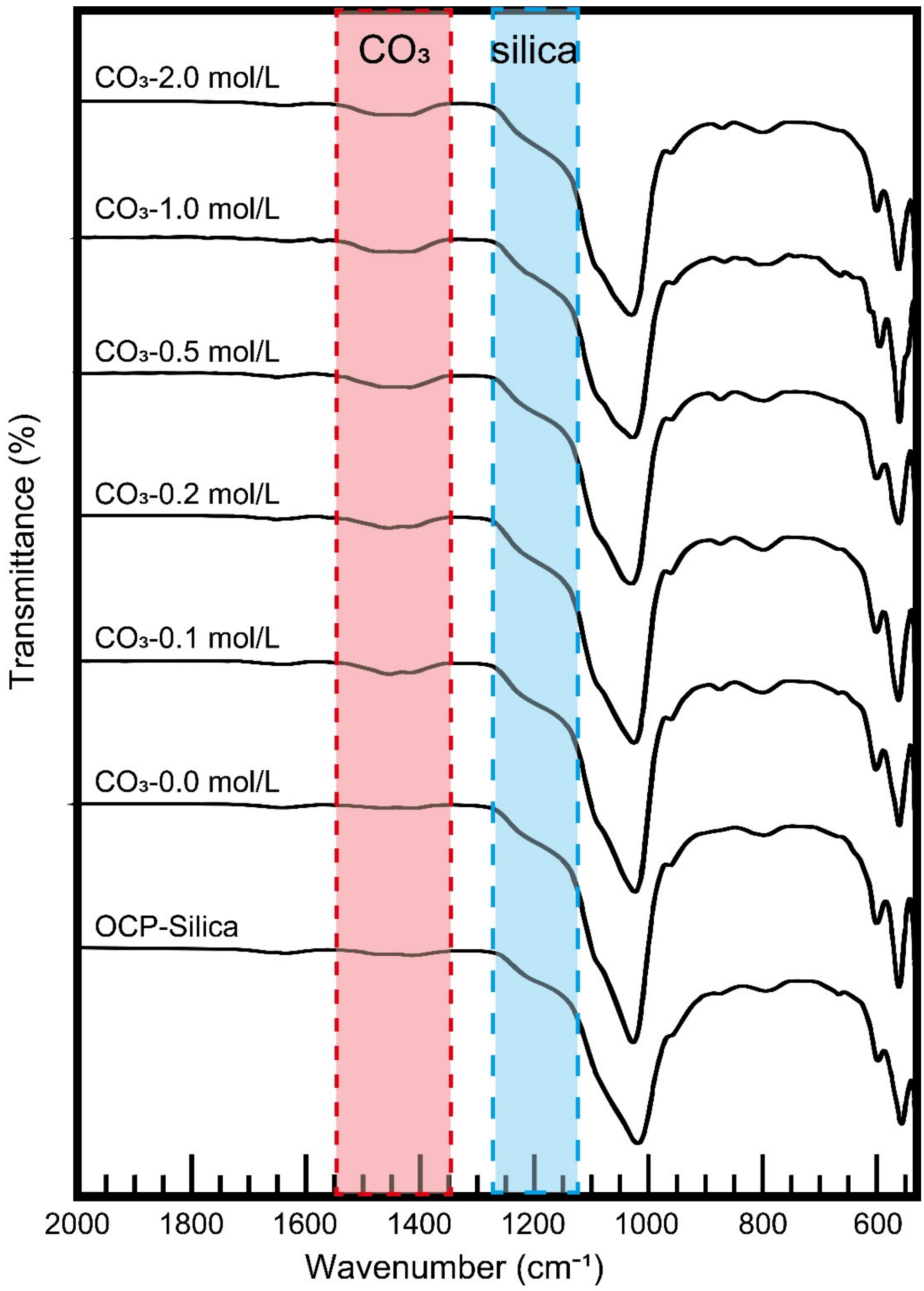

Figure S2. FT-IR spectra of OCP-silica treated in various $(NH_4)_2CO_3$ solutions.

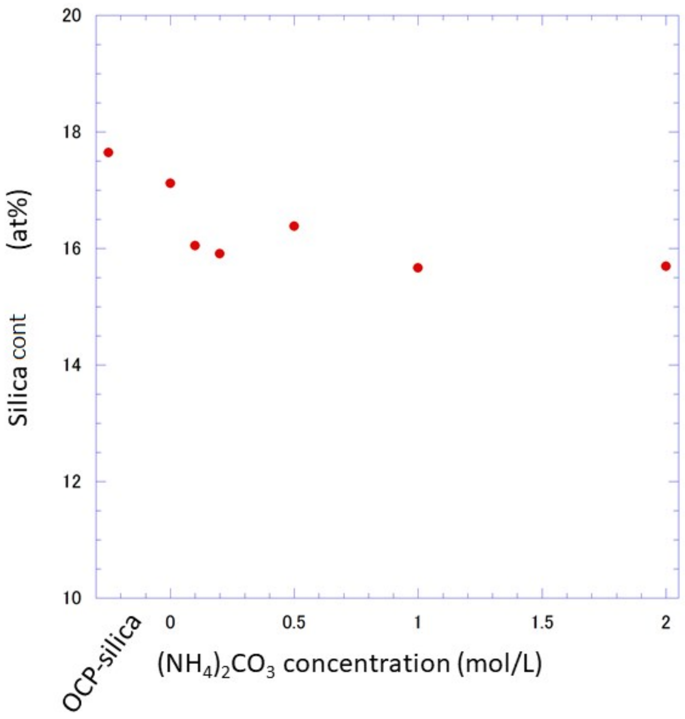

Figure S3. Silica contents of OCP-silica treated in various $(NH_4)_2CO_3$ solutions.

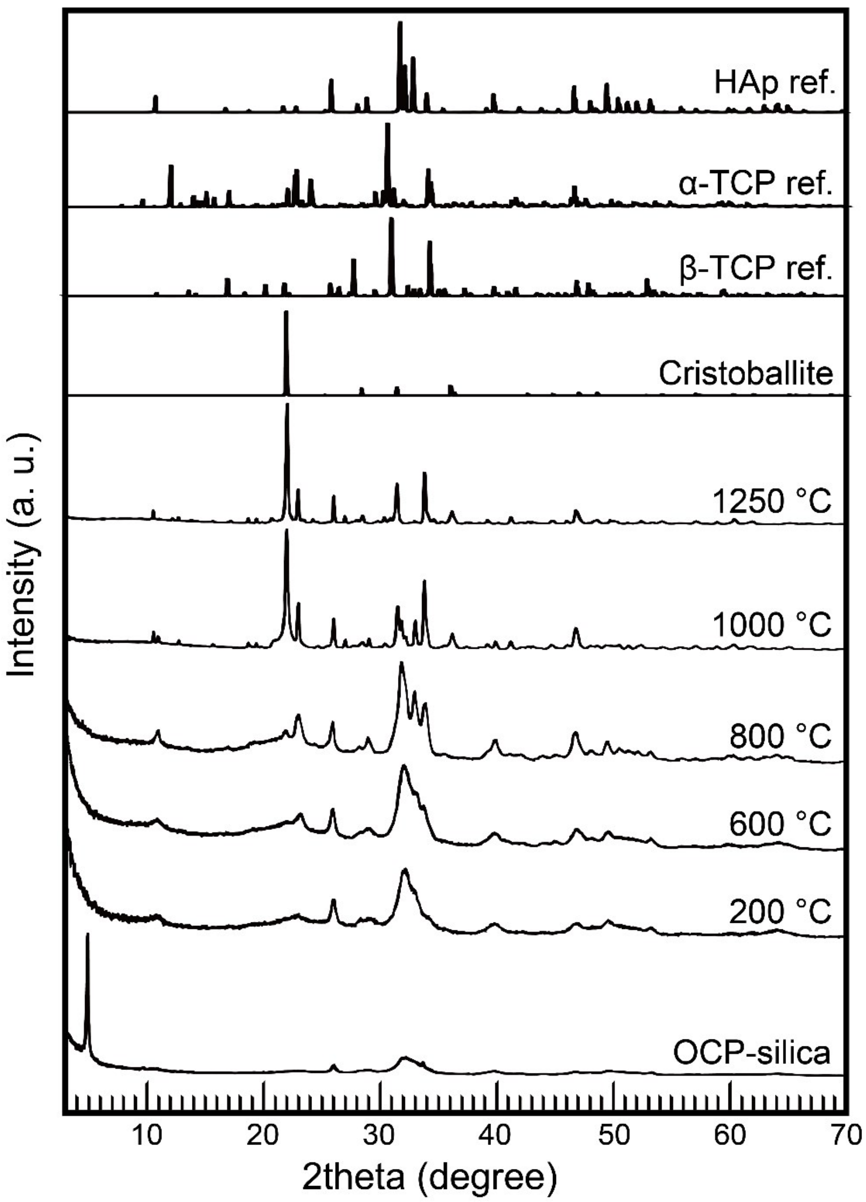

Figure S4. XRD patterns of OCP-silica subjected to heat treatment.

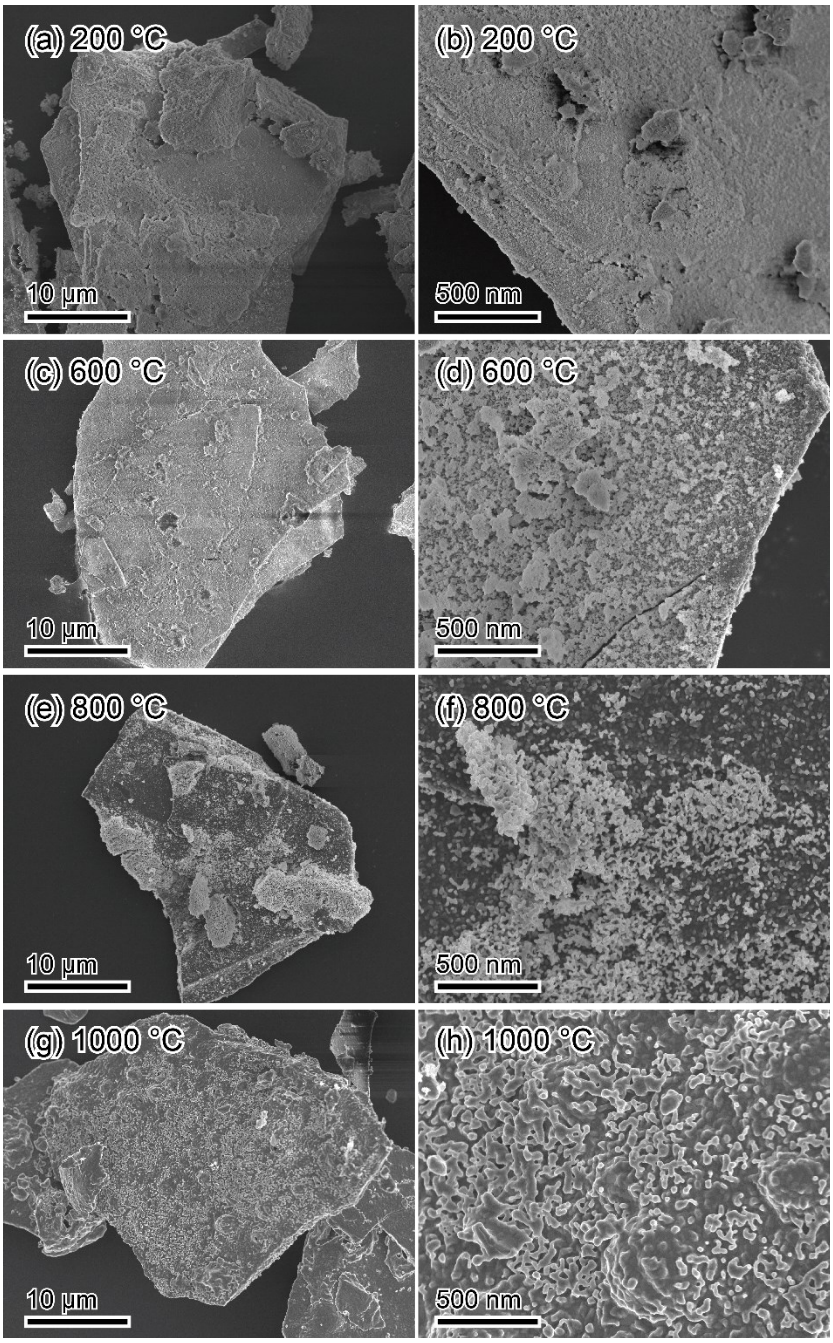

Figure S5. SEM micrographs of OCP-silica subjected to heat treatment. (a,b) 200 °C. (c,d) 600 °C. (e,f) 800 °C. (g,h) 1000 °C.

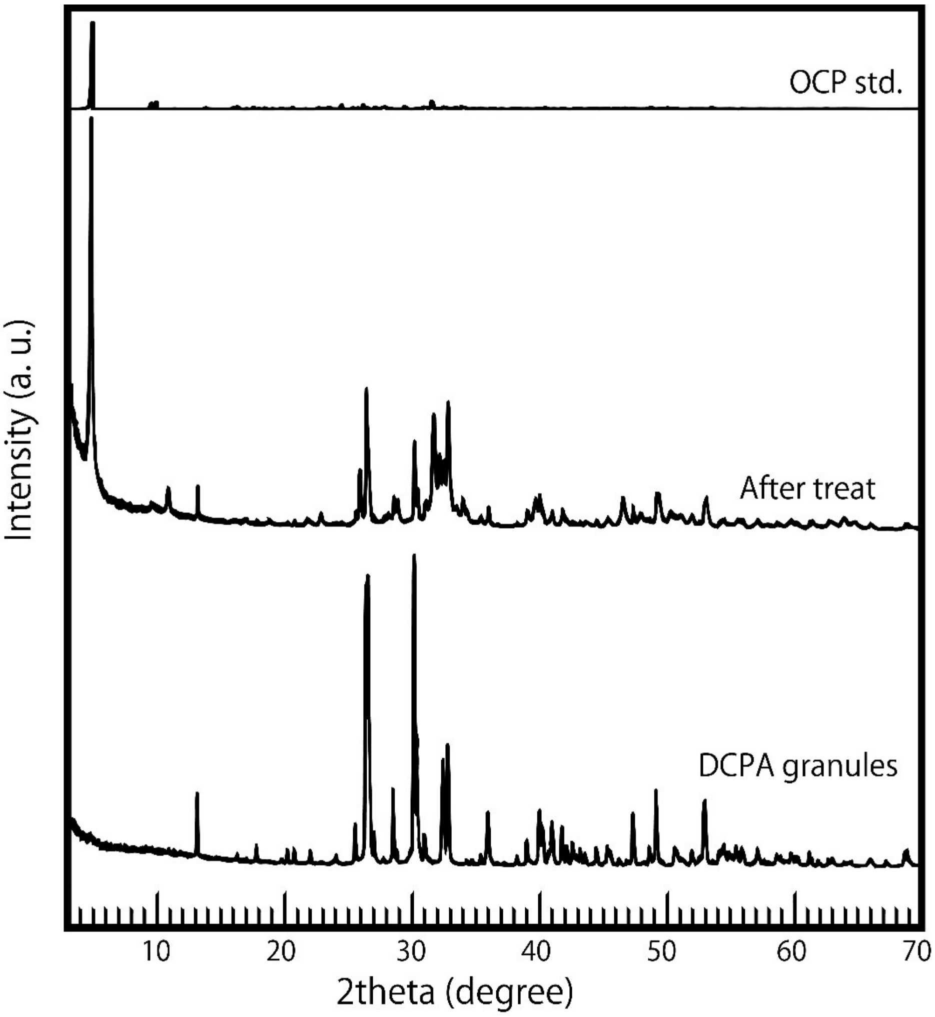

Figure S6. XRD patterns of DCPA granules before and after treatment in 1 mol/L Na_2SiO_3.

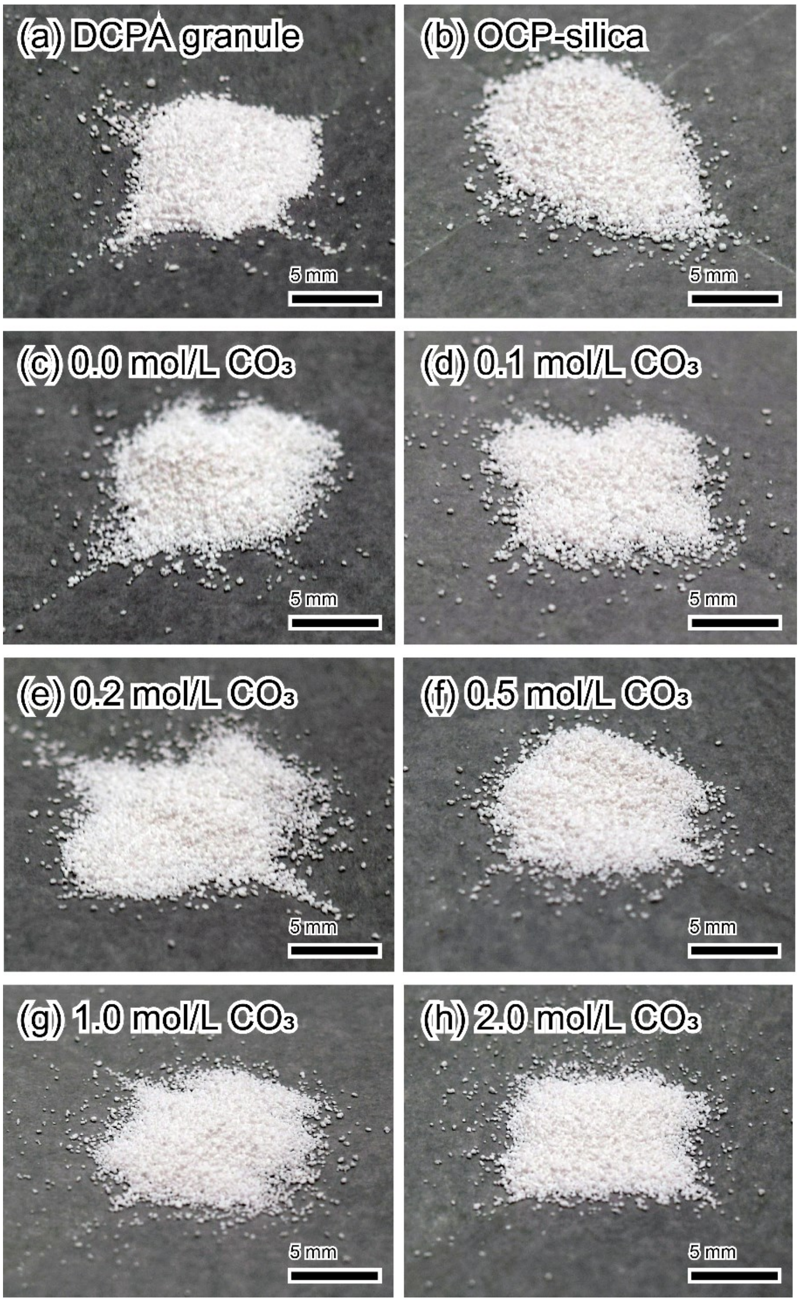

Figure S7. Photographs of (a) DCPA granules, (b) OCP-silica granules, and OCP-silica granules treated in (c) H_2O, (d) 0.1 mol/L, (e) 0.2 mol/L, (f) 0.5 mol/L, (g) 1.0 mol/L, and (h) 2.0 mol/L $(NH_4)_2CO_3$ solutions.

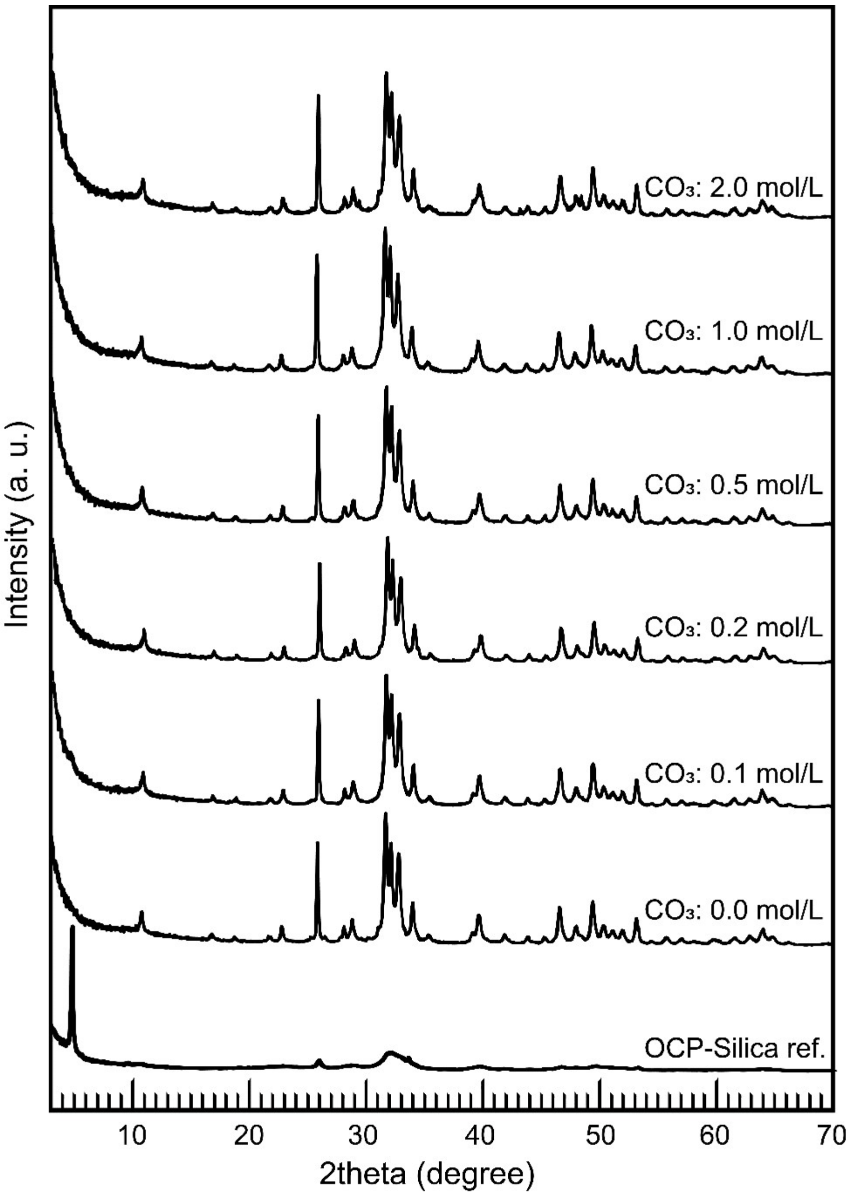

Figure S8. XRD patterns of OCP-silica granules treated in various $(NH_4)_2CO_3$ solutions.

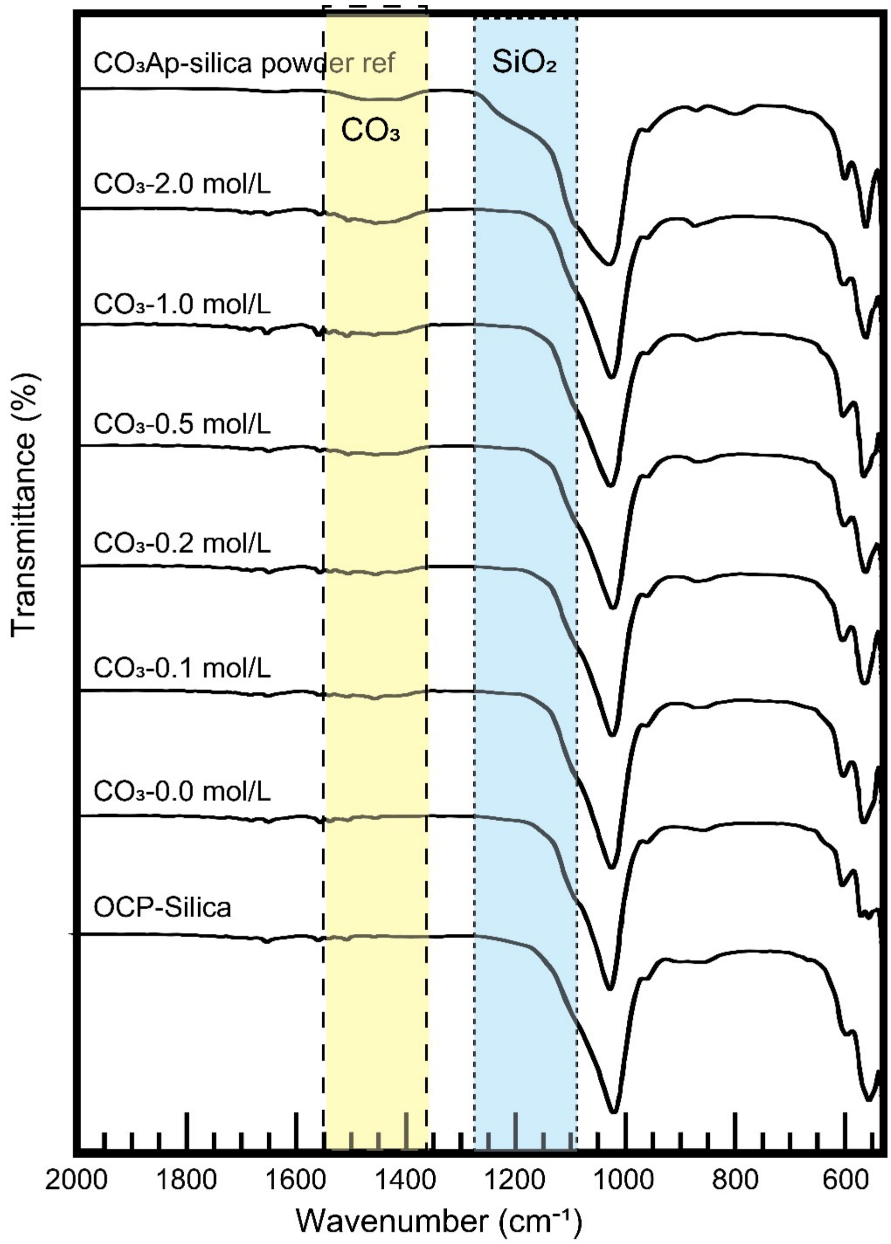

Figure S9. FT-IR spectra of OCP-silica granules treated in various $(NH_4)_2CO_3$ solutions.
